# Re‐interpreting tumour behaviour and the tumour microenvironment as normal responses to tissue disorganisation

**DOI:** 10.1002/path.6070

**Published:** 2023-03-28

**Authors:** Paul AW Edwards

**Affiliations:** ^1^ Department of Pathology University of Cambridge Cambridge UK

**Keywords:** tumour microenvironment, wound healing, epithelial–mesenchymal transition, cancer‐associated fibroblast, tumour‐infiltrating lymphocytes, malignancy, cancer

## Abstract

Much of tumour cell biology and the tumour microenvironment may be normal wound‐healing responses as a *consequence* of the disruption of tissue structure. This is why tumours resemble wounds, and many features of the tumour microenvironment, such as the epithelial–mesenchymal transition, cancer‐associated fibroblasts, and inflammatory infiltrates, may largely be normal responses to abnormal tissue structure, not an exploitation of wound‐healing biology. © 2023 The Author. *The Journal of Pathology* published by John Wiley & Sons Ltd on behalf of The Pathological Society of Great Britain and Ireland.

## Introduction

Much of contemporary cancer research is focused on the cell biology of tumour cells and their surrounding stroma – on phenomena such as the ‘epithelial–mesenchymal transition’ (EMT), ‘cancer‐associated fibroblasts’ (CAFs), and ‘tumour‐infiltrating lymphocytes’ (TILs) [1, 2, 3], which together form the ‘tumour microenvironment’. I suggest that much of this biology may be understood as the inevitable and normal consequence of the disruption – i.e. wounding – of the epithelium or other tissue from which the tumour developed, and is not a ‘deliberate’ exploitation of these behaviours for the tumour's benefit as previously suggested [4].

This may be an example of a recurring problem in cancer research, of finding interesting phenomena in tumours without realising that they are normal phenomena (see Box 1).

Box 1 **Mistaking normal cell properties for tumour‐specific properties.**

The difficulty of separating tumour behaviour from normal behaviour has a long history in cancer research. Over 100 years ago, Peyton Rous [5] (of Rous sarcoma virus) warned of this: “essential … in cancer work to discriminate between characters unique with tumour and those which it possesses in common with normal tissue.” He was criticising Ehrlich, who had found that tumours grafted from one mouse to another were rejected, but who had not done the control of grafting normal tissue, which, in the outbred mice he used, would also have been rejected. Ehrlich had in fact discovered the rejection of grafts between non‐identical animals (allografts). More recent examples include the discovery of candidate ‘leukaemia‐specific antigens’, which turned out to be normal stem‐cell markers [6]; and the finding that surface antigens on epithelial cancers were heterogeneously expressed, which was at first interpreted as revealing tumour heterogeneity but which proved to be typical of normal epithelia [7].

This reflects a general principle that tumours do not invent new biology: they have lost or altered control of pre‐existing mechanisms. Also, behaviours seen in tumours, such as fibrosis, are more likely to be ‘normal’ responses than tumour‐specific host defences [4].

## Cancer tissue is wounded tissue

**Figure 1:**
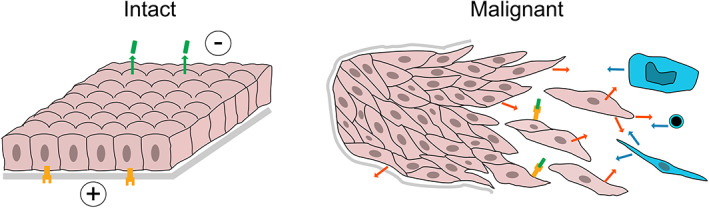
Epithelial tumours viewed as wounded epithelia. Epithelial tumours are disrupted epithelium, and will therefore mount at least some of the responses that a normal epithelium would when disrupted. Left: intact epithelium is a continuous sheet with an electrical potential across it [8], salt differences (not shown), and ligands produced at one face (shown green on apical face) that cannot reach receptors on the other face (shown yellow on basolateral face) [9]. Grey, basement membrane. Right: in malignant epithelial tumours, the epithelial sheet is disrupted, and the basement membrane may or may not be breached [10]. This activates wound‐healing responses in the epithelium, which include signals (orange arrows) to stromal cells, represented to the right, in blue, by a macrophage, lymphocyte, and fibroblast; signals from the alerted stroma back to the epithelium (blue arrows); electric currents; and interaction between ligands and receptors [9, 11].

Cancer can be viewed as a failure of the 3D structure of tissues – the failure of normal tissue development, homeostasis, and/or the ability to repair damage through regeneration and wound healing. This is easiest to understand for epithelial tumours: normal epithelial cells are part of a sheet, with an inside and outside, and a salt difference and electrical potential between the inside and outside (Figure 1). Disruption of the sheet results in alarm signals to trigger repair and regeneration: these signals probably include collapse of the trans‐epithelial electrical potential [8, 11], receptors at one epithelial face being exposed to ligands secreted from the other face (e.g. the HER/EGFR/ERBB family on the basolateral face and NRG1/heregulin on the apical face) [9], and changes in cell shape and tension [11]. The adjacent stroma probably also responds: to signals from the distressed epithelium, or to exposure to epithelial cells without a separating basement membrane [12]. The stroma will, in turn, signal back to the epithelial cells (Figure 1) [11].

Malignant epithelial tumours are chaotic mixtures of disrupted epithelium and stroma (Figure 1). The epithelium is no longer an intact sheet, and may or may not be separated from the surrounding stroma by a basement membrane [10], so the tumour and adjacent non‐tumour cells should be frantically trying to restore correct structure and will mount a wound‐healing response. Much of what we see in a tumour may be this response.

## “Tumours [are] wounds that do not heal”

Many authors have described the similarities between cancer and wound healing, down to details such as gene‐expression profiles [4, 12, 13], but, to my knowledge, where they have interpreted the similarity, they have suggested that tumours *exploit* wound‐healing programmes to achieve properties such as invasiveness, rather than the wound‐healing programme being an inevitable *consequence* of the disruption of the epithelium. In particular, Dvorak, who in 1986 proposed that a cancer is “a wound that does not heal”, also “proposed that tumours co‐opted the wound‐healing response to induce the stroma *they required*” (my italics), and “tumours disguise themselves as wounds and call upon the host to ‘heal’ them” [4]. A recent review [14], for example, states “Cancer‐associated fibroblasts (CAFs) … can adopt … [a phenotype] comparable to … fibroblasts in … wound‐healing”, and apparently does not consider that these fibroblasts are indeed wound‐healing fibroblasts because the tissue is mounting a wound‐healing response.

These contrasting concepts are not, of course, mutually exclusive: some wound‐healing responses may be consequences of disruption, while others may be invoked by cancer mutations.

## Applying the idea

Perhaps, then, we could understand the behaviour of malignant tumour cells by reference to what normal cells would be doing in similar circumstances – which we can call ‘wound healing’. In an epithelial tumour, the epithelial cells and stroma will be changing behaviour in response to breach of the epithelial sheet, and other changes in structure or behaviour.

## The ‘epithelial–mesenchymal transition’ (EMT)

The ‘epithelial–mesenchymal transition’ is a set of phenotypic changes in epithelial tumour cells towards a more mesenchymal phenotype, such as loss of polarity and expression of the mesenchymal protein vimentin [1]. It is part of normal wound healing but has, controversially, also been invoked as a mechanism – indeed a driver – of malignancy, invasion, and metastasis [1, 12].

We can re‐interpret EMT as an inevitable consequence of disrupting epithelium, not something that tumour cells exploit to invade and metastasize [1, 12].

## Motility, proteases, and angiogenesis

It is often assumed that invasion and metastasis require enhanced motility [15], but normal epithelial cells at a wound are highly motile [12] – skin keratinocytes at a wound edge can move around 50 μm/day [16], much faster than a typical tumour infiltrates tissue. The observed high motility of some tumour cells may be their normal response to disorganisation.

Epithelial wound healing also includes secretion of matrix metalloproteases to break down clot, and initiates angiogenesis [11, 12].

## Dense stroma and cancer‐associated fibroblasts (CAFs)

Fibrosis, i.e. scarring, the laying down of collagen‐rich tissue by fibroblasts, occurs at the breach of an epithelium (think of scars forming where skin is cut). The dense, collagen‐rich stroma seen in many tumours, called ‘desmoplasia’, resembles chronic fibrosis [4, 17], so could simply be the response to broken epithelium. The various fibroblast‐like cells found in tumour stroma, ‘cancer‐associated fibroblasts’, are similar to cells observed in wounds [2].

## Lymphocyte and macrophage infiltration

There is much interest in inflammatory cells in tumours, notably ‘tumour‐infiltrating lymphocytes’ (TILs), because of their possible role in an immune response to the tumour [3]. But any breach in an epithelium threatens infection, so some of the inflammatory cells (neutrophils, macrophages, or lymphocytes) could be summoned to defend against infection, rather than the tumour. Indeed, some TILs are primed to viruses that infect epithelia [18].

## Differences and variability

Changes seen in tumours will not, of course, exactly match those in a wound caused by mechanical damage. For example, tumours do not usually cause haemorrhage, which contributes to damage signalling via complement and platelet activation [11]. And matrix structures such as basement membranes may or may not be disrupted [10].

The tumour microenvironment will also vary between tumours because of variations in their growth pattern, anoxia, and necrosis, etc. (as well as any differences in mutations that affect wound healing). For example, lobular breast cancer cells invade in single file, while many other breast cancers are solid masses of cells.

## Why is this a useful argument?

This line of argument may be useful because it may provide a framework for understanding tumour behaviour. For example, cancer‐associated fibroblasts are probably best understood by comparison with fibroblasts in wounds, as already suggested [2]. We might better understand the role of EMT in invasion if we knew whether there were differences between EMT in wound healing and in cancers.

This argument should also help us to understand how mutations lead to cancer, by separating out the components of tumour behaviour that are truly abnormal, and hence the product of mutation. This would also inform therapy, which might otherwise target components of normal wound healing without much benefit to the patient.

It also generates new questions and redirects old ones. A recurring question about the similarity between tumours and wounds has been why wound‐associated changes are transient in wounds but persist in tumours (although reversing malignancy can reverse the changes [19]). This becomes the wrong question if changes are caused by disorganisation, which persists.

This argument also focuses attention on what drives disorganisation, and, more precisely, how mutations disrupt organisation. Is unlimited survival and proliferation alone enough to bulldoze through normal homeostasis and disrupt tissue architecture, or does malignancy also require alteration of signals that control 3D organisation?

An important question is whether cancer driver mutations directly modify the wound‐healing response, for example by enhancing EMT or sending signals to the stroma. This seems likely. A component of EMT is down‐regulation of E‐cadherin/*CDH1*, and inactivation of *CDH1* is a driver mutation, for example in lobular breast cancer. Oncogenic signalling pathways including the Wnt, TGF‐β, and Notch pathways apparently directly affect EMT, even in normal tissues [20]. Activating *KRAS* can modify the ability of epithelial cells to correct tissue damage [21]. But whether driver mutations directly modify stromal responses is more difficult to pin down, because we must distinguish between direct effects and the indirect effects of breaking the rules of tissue organisation. Nevertheless, it seems that oncogenic mutations can cause inflammatory signalling [22]. For example, activating *KRAS* in pancreatic epithelium can reprogramme neighbouring fibroblasts [23], and activated *RAS* genes can induce senescence, which results in signalling to neighbouring cells [24].

Dvorak's [4] original concept, that tumours exploit wound healing, could still prove to be insightful. Could turning on wound‐healing responses permit cells to proliferate and move into inappropriate environments? Could, for example, the constitutive activation of Erbb2/Her2 seen in some carcinomas be mimicking the proliferative wound signal, access of apical Nrg1 ligand to basolateral Erbb2 [9]?

## Conclusion

In conclusion, we need to know how much of malignant tumour cell biology is simply programmed in as a normal wound‐healing response, consequent on disruption of normal tissue architecture. And we need to understand how cancers become disorganised, in fact how they become malignant.
